# A framework using topological pathways for deeper analysis of transcriptome data

**DOI:** 10.1186/s12864-019-6155-6

**Published:** 2020-03-05

**Authors:** Yue Zhao, Stephanie Piekos, Tham H. Hoang, Dong-Guk Shin

**Affiliations:** 1 Computer Science and Engineering Department, University of Connecticut, 371 Fairfield Way, Unit 4155, Storrs, 06269 USA; 2 Department of Pharmaceutical Sciences, University of Connecticut, 69 North Eagleville Road, Unit 3092, Storrs, USA

**Keywords:** Topological Pathway Analysis, Bayesian Network, Depth First Search

## Abstract

**Background:**

Pathway analysis is one of the later stage data analysis steps essential in interpreting high-throughput gene expression data. We propose a set of algorithms which given gene expression data can recognize which portion of sub-pathways are actively utilized in the biological system being studied. The degree of activation is measured by conditional probability of the input expression data based on the Bayesian Network model constructed from the topological pathway.

**Results:**

We demonstrate the effectiveness of our pathway analysis method by conducting two case studies. The first one applies our method to a well-studied temporal microarray data set for the cell cycle using the KEGG Cell Cycle pathway. Our method closely reproduces the biological claims associated with the data sets, but unlike the original work ours can produce how pathway routes interact with each other above and beyond merely identifying which pathway routes are involved in the process. The second study applies the method to the p53 mutation microarray data to perform a comparative study.

**Conclusions:**

We show that our method achieves comparable performance against all other pathway analysis systems included in this study in identifying p53 altered pathways. Our method could pave a new way of carrying out next generation pathway analysis.

## Background

In this era of biomedical big data, a noticeable trend is that newly acquired genomics data (specifically, gene expression data) is compared with the prior known gene regulation relationships which are typically organized into curated molecular pathways (e.g., KEGG [1], Biocarta [2], Reactome [3], Wikipathways [4]). In general, gene expression data is first processed to identify significant differentially expressed (DE) genes using statistical methods like Limma [5], SAM [6], SPH [7], etc. These identified DE genes are then divided into groups of similar patterns using clustering programs [8] or pattern based programs [9, 10]. Each group of similarly behaving genes is then examined to test if each group includes genes known for any particular biological function (e.g., GOStat [11]) or molecular pathway at unusually high frequencies (e.g., DAVID [12], GSEA [13]). Although these gene enrichment analysis methods are useful in recognizing some basic nature of perturbed signals of the biological system under study, they do not discern if any specific pathway is *activated* or *suppressed* other than the fact that some pathway could be highly involved in the experimental system being studied. The next generation pathway analysis methods aimed at overcoming such deficiency of gene enrichment methods by organizing known gene-gene interaction relationships into topological pathways and analyze gene expression data on top of them so that the activated or suppressed state of the pathway can be computationally revealed (e.g., PARADIM [14], SPIA [15]).

We previously published topology-based pathway analysis methods belonging to this next generation pathway analysis system [16–19]. Specifically, our method presented in [18, 19] departs from the conventional topology-based systems like PARADIM or SPIA in the sense that our method dynamically encodes pathway routes as a Bayesian network and uses both gene expression and mutation data as input and identifies not only if any pathway is activated or suppressed but also through which *route(s)* of the pathway such gene expression perturbation could be propagating. However, one limitation of our previous work is that the method requires preselection of the start and end of pathway routes to be analyzed. In addition, through empirical studies, we discover that our previous method tends to identify “choppy” pathway routes that are partially activated or suppressed, thus less useful if one’s goal is to find overall patterns of pathway route usages. The goal of this paper is to report the extension of our previous work [18, 19] in which multiple new algorithms are introduced to isolate highly regulating (activation and/or suppression) sub-components of the pathways and conveniently visualize the overall patterns of pathway activation or suppression directly over the pathway diagrams. We call this system Deep Pathway Analyzer (DPA).

Among existing gene set enrichment analysis methods, GSEA is one of the most popular software packages in which computing the enrichment score is done by a variation of the weighted Kolmogorov-Smirnov-like statistic [13]. SPIA by [15] is a topology-based system and it proposes to measure pathway significance by performing statistical tests against random permutation. An improvement over SPIA is PARADIGM [14] which models the pathway as a factor graph and uses a statistical method to compute a sample specific inference, specifically for genomics data obtained from cancer patients. Two recent systems by [20] and [21] also encode the pathway as a Bayesian network. After removing cycles in the graph, they train the model with expression data. Significance of the score is produced by bootstrap-generated data. DRAGEN by [22] detects differentially expressing genes by performing a hypothesis testing designed to figure out if linear model has identical parameters. Most recently, Altered Pathway Analysis tool (APA) by [23] aims to detect altered pathways by dynamically calculating pathway rewiring through analyzing correlation between genes, but this system does not use prior knowledge. Our work is different from these existing topology-based systems by the feature, what we call, route-based recognition capability, and using this feature we can produce deeper analysis outcomes suggesting how identified "perturbed" pathway routes may interact with each other.

The rest of this paper is organized as follows. “Methods” section briefly reviews existing pathway analysis methods and introduces the Bayesian network model and the algorithms newly developed. Section III describes the results of our algorithm being tested using a public domain temporal microarray data set from the cell cycle experiment [24]. Afterwards we show the outcome of applying our algorithm to the p53 mutation microarray data and specifically compare our analysis outcome with the similar analysis done by [23]. Two case studies are shown to demonstrate the generality of our enhanced method. Lastly, Section VI is the conclusion.

## Methods

In this section we first briefly review the methodology proposed in our previous work [19] for the sake of completeness and then present two new algorithms that are designed to improve deficiencies of the earlier system.

### Review of the previous Model

The key idea of DPA is identifying “routes” of aberrant pathways. Each pathway route *G*^∗^ is encoded as a Bayesian Network *G* which is initialized with a sequence of conditional probabilities which are designed to encode directionality of regulatory relationships encoded in the pathways, i.e., activation and inhibition relationships. The transformation process from *G*^∗^ to the corresponding Bayesian Network *G* is illustrated in Algorithm.1. Next we show the biological interpretation logic behind the conditional probability table for *e*_*ij*_. Consider the activation table given in (Table.1) (for the inhibition table, refer to (Table 2) which is built in a similar way): If the parent gene of *g*_*j*_, *g*_*i*_, has function gain mutation, and overly expressed, namely *M*_*i*_=*R*_*i*_=+1, then the target *g*_*j*_ would also be highly likely to overexpress, i.e. *R*_*j*_=+1, given the edge between them in *G*^∗^ is ‘activation’. As a result,

$$P(R_{j}=+1|M_{i}=R_{i}=+1)=1-\epsilon $$

where *ε* is the error rate we can tolerate and is close to zero. Similarly, if the parent gene of *g*_*j*_ has function loss mutation, or its expression level is down-regulated in test case, then the downstream regulation towards *g*_*j*_ would be likely not functioning. Therefore, *g*_*j*_ would tend to be underexpressive, namely *R*_*j*_=−1, and the corresponding probability is flipped.

**Table 1:** Activation

*M*_*i*_	*R*_*i*_	*R*_*j*_=+1	*R*_*j*_=−1
+1	+1	1−*ε*^∗^	*ε*
−1	+1	*γ*	1−*γ*
+1	−1	1−*γ*	*γ*
−1	−1	*ε*	1−*ε*

**Table 2:** Inhibition

*M*_*i*_	*R*_*i*_	*R*_*j*_=+1	*R*_*j*_=−1
+1	+1	*ε*	1−*ε*
−1	+1	1−*γ*	*γ*
+1	−1	*γ*	1−*γ*
−1	−1	1−*ε*	*ε*

^∗^*ε*∈(0,0.5) is the error rate we could tolerate

Let the pathway of interest be converted into a gene regulation network *G*_*B*_=(*V*_*B*_,*E*_*B*_), where *V*_*B*_={*g*_*i*_|*i*=1…|*V*_*B*_|} and *E*_*B*_={(*g*_*i*_,*g*_*j*_)|*g*_*i*_,*g*_*j*_∈*V*_*B*_}. Consider a given pathway route *G*^∗^=(*V*^∗^,*E*^∗^) in *G*_*B*_ where $V^{*}=\{g_{i_{k}}|k=1\ldots |V^{*}|\}$ and *E*^∗^={(*g*_*i*_,*g*_*j*_)|*g*_*i*_,*g*_*j*_∈*V*^∗^}⊂*E*_*B*_.

Once the Bayesian Network *G* is generated from *G*^∗^, the pathway route is ranked by conditional probability of the observed data given *G* normalized by *P*(**R**,**M** are consistent|*G*) as shown in (1) [19] where **r**_*s*_, **m**_*s*_ are, respectively, the expression observation and the mutation observation for the sample *s*.

Advantages of this measure are: (i) the analysis could allow biologists to easily pinpoint which biological processes are likely to be overly activated or suppressed; and (ii) even though some expression values are flipped due to random errors from the genomic data (it is observed to be −1 when it is actually +1), the whole path would still have a high score since the majority of other genes could have consistent expression observations.

1 $$  Score(G^{*},\mathbf{r}_{s},\mathbf{m}_{s}) = \frac{P(\mathbf{R}=\mathbf{r}_{s},\mathbf{M}=\mathbf{m}_{s}\mid G)} {P(\mathbf{R},\mathbf{M}\text{ are consistent}\mid G)}  $$

THE REGULATION PROCESS FOR *e*_*ij*_ IN *G*^∗^

Then the score is extended to be a signed score by (2) which varies from −1 (highly suppressed) to +1 (highly enhanced). The definition of a pathway route being “activated” or “suppressed” is the following.

2 $$  \begin{aligned} sScore(G^{*},\mathbf{r}_{s},\mathbf{m}_{s})=&\tilde{I}(r_{s}^{|G^{*}|},\dot{r}_{s}^{|G^{*}|})\cdot Score(G^{*},\mathbf{r}_{s},\mathbf{m}_{s})\\ \tilde{I}(x,y)=&\begin{cases} +1 & x=y \\ -1 & x\neq y \end{cases} \end{aligned}  $$

where $r_{s}^{|G^{*}|}$ is the observed expression level of the last available node for the input sample *s* in the route *G*^∗^ and $\dot {r}_{s}^{|G^{*}|}$ is the expected expression level of the same node calculated by the interpretation logic.

Aggregating the scores for routes in a pathway, we define the pathway score in (3). We simply measure the significance of this pathway, *G*_*B*_, by using the proportion of routes that have an average of all the patients’ scores, calculated by equation (1), that is larger than some threshold *t*. Each perturbed route is weighted by its length.

3 $$ {{}\begin{aligned}  pScore_{S}(G_{B})&=\frac{1}{\sum_{G^{*}\in G_{B}}w_{G^{*}}}\\ &\sum_{G^{*}\in G_{B}}w_{G^{*}}I(\frac{1}{|S|} \sum_{s\in S}Score_{s}(G^{*})\geq t) \end{aligned}}  $$

### Statistical Significance Measure on the Route Score

In this section, we introduce a new measure to quantify the statistical significance for the route score: the probability of route score being one in (1) conditioning on the observation for each gene in route *G*^∗^ being randomly generated. The formula is shown in (4). Mutation data *m*_*s*_ is sparse and the probability of observing given *m*_*s*_ by chance is close to zero, thus it is not proper to consider the randomness of *m*_*s*_ here. Based on this assumption, *m*_*s*_ is treated as prior parameter. Thus the score is reduced to (5).

4 $$  \begin{aligned} SigScore(G^{*},\mathbf{r}_{s},\mathbf{m}_{s})=&P(\mathbf{r}_{s},\mathbf{m}_{s}\text{ are consistent}\mid P_{0})\\ P_{0}: R=&\begin{cases} +1& p=0.5\\ -1& p=0.5 \end{cases} \end{aligned}  $$

5 $$ {{}\begin{aligned} SigScore(G^{*},\mathbf{r}_{s},\mathbf{m}_{s})=P(\mathbf{r}_{s}\text{ are consistent}\mid P_{0}, \mathbf{M}_{s}=\mathbf{m}_{s}) \end{aligned}}  $$

Suppose *w* is the number of genes in the route, then

$${{}\begin{aligned} SigScore(G^{*},\mathbf{r}_{s},\mathbf{m}_{s})\,=\,&P(\mathbf{r}_{s}\text{ are consistent}\mid P_{0},\mathbf{M}_{s}=\mathbf{m}_{s})\\ =& 2 (0.5)^{w}= (0.5)^{w-1} \end{aligned}} $$

In order to measure the significance of the pathway score in (3), we calculate the probability of observing *Q* differentially regulated routes in a pathway *G*_*B*_ given the observations are selected randomly. The number *Q* follows *Poisson* *Binomial* *Distribution* [25] and this probability can be approximated by (6) [26] assuming *G*_*B*_ consists of *k* routes.

6 $$  {{}\begin{aligned} SigScore_{G_{B}} = \Pr(Q = q) \approx&\ \text{Binom} \left(n, \frac{\mu}{k} \right)\\ \mu =& \sum_{G^{*}\in G_{B}} \prod_{s} SigScore(G^{*},\mathbf{r}_{s},\mathbf{m}_{s}) \end{aligned}}  $$

This probability can serve as the p-value of the hypothesis test whose null hypothesis is that the observation is generated randomly by *P*_0_. Thus low $SigScore_{G_{B}}$ indicates rejection of null hypothesis, and the lower the SigScore is, the more significant the calculated pathway score is.

### Hyper Parameter Analysis and Dynamic Parameter Setting

In this section, we discuss issues related to setting the hyper-parameters. The key idea behind setting the hyper parameters is to make the false discovery rate associated with route score controllable. Consider a pathway route *G*^∗^ with length of |*G*^∗^| having |*G*^∗^|−1 edges. The route score in (4) can be approximated by a simpler formula (7) involving the number of inconsistent edges, *K*. This formula is to capture the intuition that whenever an inconsistent edge is discovered according to data, we penalize the score by the hyper-parameter *ε*, and otherwise we reward the score by 1−*ε*.

7 $$  \begin{aligned} SigScore(G^{*},K)=\frac{\epsilon^{K}(1-\epsilon)^{|G^{*}|-1-K}}{(1-\epsilon)^{|G^{*}|-1}}=(\frac{\epsilon}{1-\epsilon})^{K} \end{aligned}  $$

Next we proceed to derive the distribution of *K* assuming that each edge is discovered inconsistent independently by chance. That is, for each edge *e*_*i*_,*i*=1…|*G*^∗^|−1, we define Bernoulli random variable *X*_*i*_=1 if *e*_*i*_ is inconsistent; *X*_*i*_=0 otherwise. Thus *P*(*X*_*i*_=1)=0.5, *X*_*i*_ *i*.*i*.*d*.∼*Bernoulli*(0.5). Then $K=\sum _{i} X_{i}$ follows Binomial distribution *Bin*(0.5,|*G*^∗^|−1) since *X*_*i*_’s are independent. Based on this distribution, one can pick *ε* such that the majority of the scores generated under null hypothesis (the portion ≥1−*α*) is less than some threshold *t*, namely, *P*(*SigScore*(*G*^∗^,*K*)≥*t*)<*α*. Since SigScore in (7) is monotonically decreasing by *K*, we have

8 $$  P((\frac{\epsilon}{1-\epsilon})^{K}\ge t)<\alpha  $$

$$P(K\le (\log\frac{\epsilon}{1-\epsilon})^{-1}\log t)<\alpha \ \ \forall \epsilon \in (0,0.5) $$

**Fig. 1:**
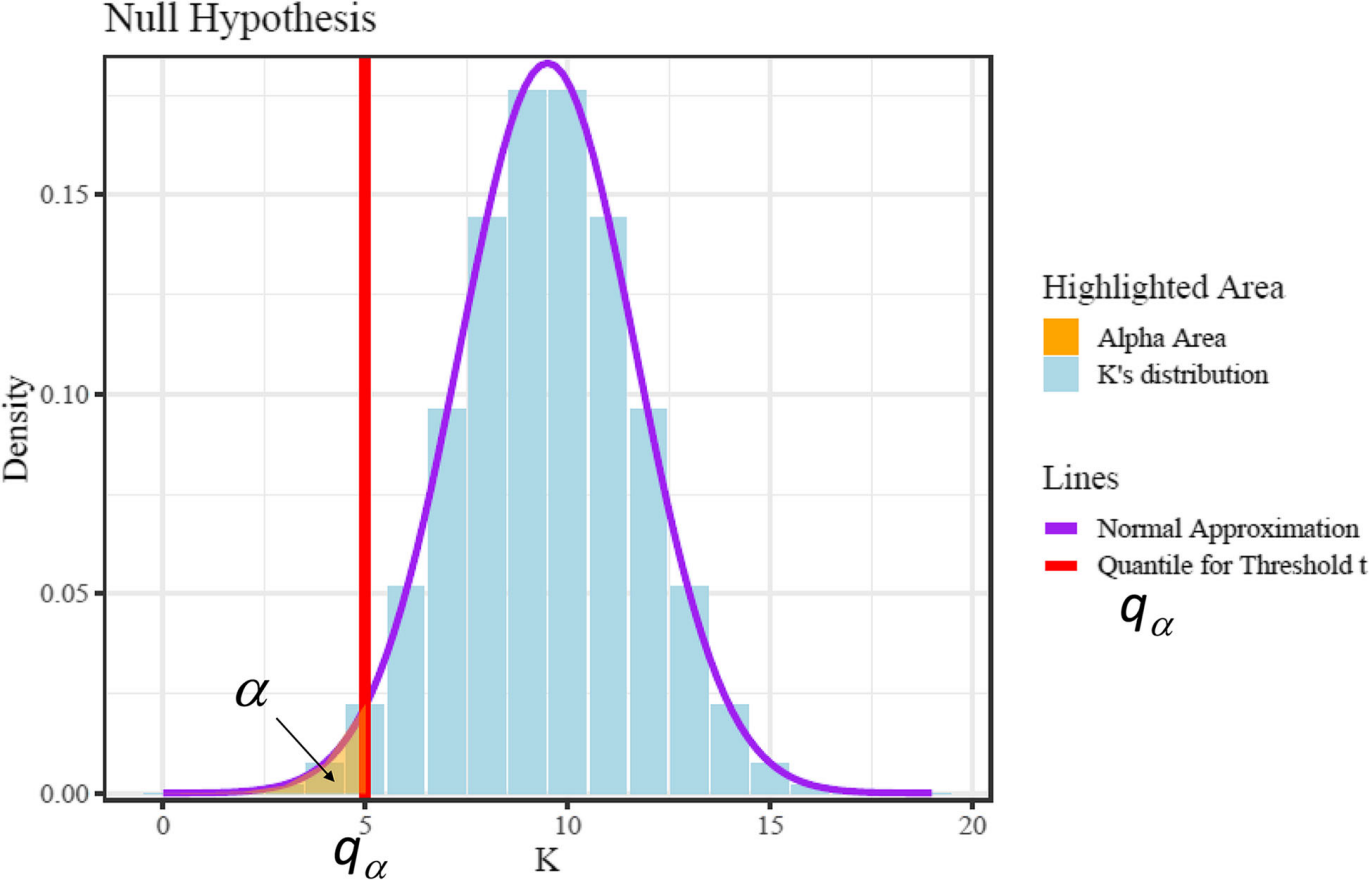
Distribution of *K*, the number of the edges being penalized, confidence level *α* and quantile for threshold *t*

Since *K*’s distribution is known, we can assign $(\log \frac {\epsilon }{1-\epsilon })^{-1}\log t$ to be no larger than *q*_*α*_, which is exactly the quantile value such that *P*(*K*≤*q*_*α*_)=*α* as shown in Fig. 1. The quantile value is available from the binomial probability table. Actually since binomial distribution offers limited confidence level options, one can use the quantile of the normal approximation of the binomial distribution instead [27]. By solving the equation, we have:

$$(\log\frac{\epsilon}{1-\epsilon})^{-1}\log t\le q_{\alpha} $$

Here when *t*=1, *K*≡0 (entirely consistent) should hold according to the equation (8) since *ε*∈(0,0.5). This indicates that independent of *ε*, only routes with no inconsistency are discovered if *t*=1, but this arrangement would be too strict.

In case when *t*∈(0,1),

9 $$  \epsilon\le B(t,\alpha)=1-\frac{1}{1+\sqrt[q_{\alpha}]{t}} \in (0,0.5)  $$

The intuition behind this formula is the following. The formula (9) clearly indicates that the upper bound of *ε*, *B*(*t*,*α*), increases if either *t* or *α* increases. If we keep the upper bound fixed, increasing *t* will make *α* decrease while providing a better confidence level and thus resulting in a smaller false positive rate.

As far as the hyper-parameter *γ* is concerned, given any edge *e*_*ij*_ in the route, the marginal probability $P(R_{j}=+1|R_{i}=+1)=\sum _{M_{i}}P(R_{j}=+1|R_{i}=+1,M_{i})/P(R_{i}=+1)=(1+\gamma -\epsilon)/2$ if no mutation information is available. To penalize inconsistency, one can set *γ* larger than *ε*. However, the inconsistency should also be penalized if mutation information is present as shown in Table 1-2, and in that case *γ* decreases making *γ*∈(*ε*,0.5). This also explains why the setting *ε*=0.1 and *γ*=0.25 gives a good result as such outcome has been presented in one of our previous works [18]. In this paper, we choose to set *γ* to be the midpoint between *ε* and 0.5, namely, (*ε*+0.5)/2.

For all the experiments in this work, we dynamically calculate *ε* using the upper bound provided by (9) with threshold *t*=0.8 and *α*=0.05 so that at most 5% scores generated randomly under the null hypothesis can become larger than 0.8 as such condition is guaranteed by (8).

### Algorithmic Approach to Deeper Pathway Analysis

Here we propose a set of algorithms which aim to recognize all the “perturbed” portion of a pathway based upon input omics data which may include not only gene expression data but also mutation data. We label “perturbed” portion as the sub-network whose gene-gene interaction relationships are recognized as “perturbed” within the network topology when the input expression data is compared to the known relationships captured in the pathway network. It uses a Depth-First-Search[28] to extract all possible routes starting from a given node in the pathway and calculates the signed score using (2) at each step so that the perturbed portion, i.e., the subnetworks falling outsides of some threshold scores close to +1 for “activated” (−1 for “suppressed”) can be isolated. This process is described formally in Algorithm.2. Due to space limitation, only the algorithm calculating the “activated” portion of the pathway is shown. Identifying the suppressed portion of the pathways can be obtained by replacing line 5 in Algorithm.2 with ‘$\mathbf {if} \ r_{|G^{*}|}==-\dot {r}_{|G^{*}|}\ \mathbf {AND}\ score==1$’.

The motivations for developing the algorithm are manifold. First, the route computation of a given pathway can be done dynamically. Second, this dynamic route computation and the generation of Bayesian network real-time allows performing the analysis comprehensively but efficiently because all small sub-segments of each long pathway route are examined independently and checked if any of sub-segments exceed the thresholds for determining significantly “activated” or “suppressed”. Third, our algorithm solution is conducive to running the analysis in parallel for speed up. The complexity of examining all possible pathway sub-segments by running process *GetRoutes* with all existing nodes in the pathway (*v*_*i*_,*i*=1…|*V*_*B*_|) as starting node is exponential. But since examining each possible starting node in a given pathway is independent of each other, the |*V*_*B*_| processes can be easily parallelized. The time complexity of this algorithm is analyzed briefly. Since the algorithm runs depth first search through the pathway graph *G*_*B*_, this takes *O*(|*E*|) steps. For each step, we need to build the Bayesian Network and calculate the conditional probability (4). For *n* random variables each possibly having *d* different possible values, the calculation takes *O*(*d*^*n*^) in the worst case. However, in our application, our route setting makes *d*≤2 and *n*≤3 for each edge, meaning at most 3 nodes (*R*_*i*_,*M*_*i*_ and *R*_*j*_ for *e*_*ij*_) each having at most 2 possible values are considered. In this case, calculating the probability takes *O*(1) time. In summary, the algorithm takes linear time *O*(|*E*|).

## Results

### Cell Cycle Study

Our first experiment is to apply our algorithms to the microarray data set by [24] which aimed to compare the gene expression pattern of well-publicized cell cycling phases, G1, S, G2, and M. Our method shows — for the first time — how the involved genes are interacting with each other in each phase over the pathway topology and how that interacting pattern changes over time revealing the repeating pattern of cell cycling phases.


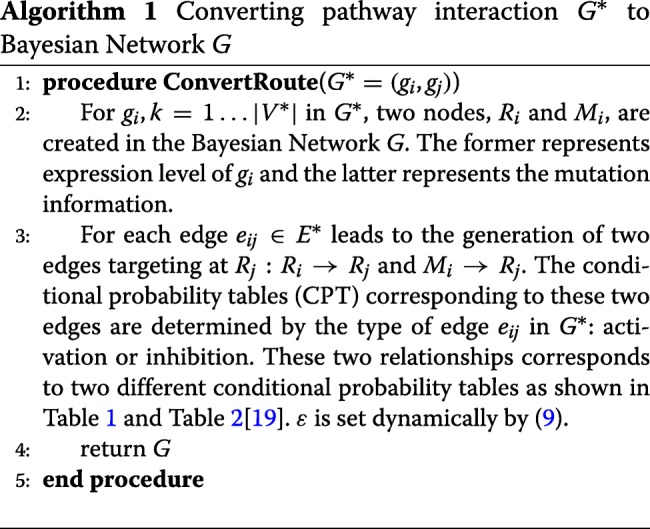


#### Data Description

The cell cycle data set by [24] used synchronized HeLa S3 cells. The microarray data was processed and log2 test-over-control RNA expression ratio was provided by the authors. We transformed the log2 ratios into expression observation **r** by (10). Log2 (Cy5/Cy3) was retrieved for each data point and used for all analyses, where (Cy5/Cy3) is the normalized ratio of the background-corrected intensities, as defined in [29]. Algorithm.2 is run with input *G*_*B*_: KEGG Cell Cycle pathway gene regulation network and *D*=(**r**,**m**). Since no mutation information is available for the S3 HeLa cell, **m** is set as a null vector. The procedure is run on all possible starting nodes in *G*_*B*_.

10 $$  r_{i}=\begin{cases} +1 & log_{2}\left(\frac{Cy5_{i}}{Cy3_{i}}\right)>0 \\ -1 & log_{2}\left(\frac{Cy5_{i}}{Cy3_{i}}\right)<0 \\ missing & otherwise \end{cases}  $$

#### Result and Discussion

**Fig. 2:**
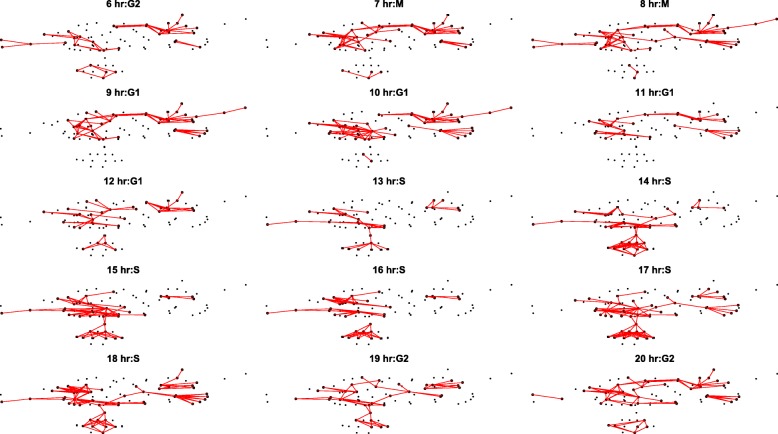
Pathway behavior by time with part of Thy-Thy Synchronization data. KEGG Cell Cycle pathway is drawn by Rgraphviz package[30]. The activated pathway routes are highlighted in red. The cell cycle phase is notated by [24]. The notation in [24] is fuzzy thus the phase on transition time point between different phases may not be completely accurate. Periodical pathway activation behavior can be observed. From the visualization, we can also see that the pathway routes has an up and down behavior pattern. And the same phase time point share similar pathway behavior as the same section of pathway gets highlighted. The biological markers for each phase are drawn as rectangles instead of circles

After extracting all possible pathway routes from KEGG Cell Cycle pathway, we calculate the scores at each time point. The network diagram shown in Fig. 2 is the Cell Cycle pathway from KEGG in which genes are displayed as nodes and prior known relationships of activation or inhibition are shown as directed edges. One important observation from this network diagram is that the changes of perturbed patterns closely match the anticipated transition of four cell cycling phases of G1, S, G2, and M as reported in the literature. The human cell cycle is a finely-tuned regulatory system consisting of multiple cellular checkpoints that allow the cell to progress through each phase, ensuring proper division. A network of proteins such as cyclins (CCNs), cyclin-dependent kinases (CDKs), and CDK inhibitors (CDKNs) regulate the cell’s transition into each phase. Changes in gene expression at the transcriptional level can be seen throughout the cell cycle, with certain genes being expressed temporally at either higher or lower levels depending on the phase of the cycle the cell is in [31]. According to the literature, mRNA levels of most of these genes correlate with their function [32]. The patterns presented in Fig. 2 could be regarded as “signatures” of pathways at different time points during the cell cycle.

Next is to report that the route scoring scheme presented by (1) successfully captures the information for each cell cycle phase. A multinomial regression LASSO model [33] is fitted to predict each cell cycle phase given the route scores calculated at different time points. By setting the penalty coefficient of 0.32, we compute the top features for each phase and the result is shown in Table 3.

**Table 3:** Top features selected for each cell cycle phase

*β*	Routes	Class
0.5596	BUB1, BUB3, ANAPC10, PTTG2, ESPL1, STAG1	M
0.3688	TFDP1, CCNE1, RB1	S
0.3222	BUB1, BUB3, ANAPC10, PTTG2, ESPL1, SMC1A	G2
0.0894	CUL1, CDKN1A, CDK6	G1


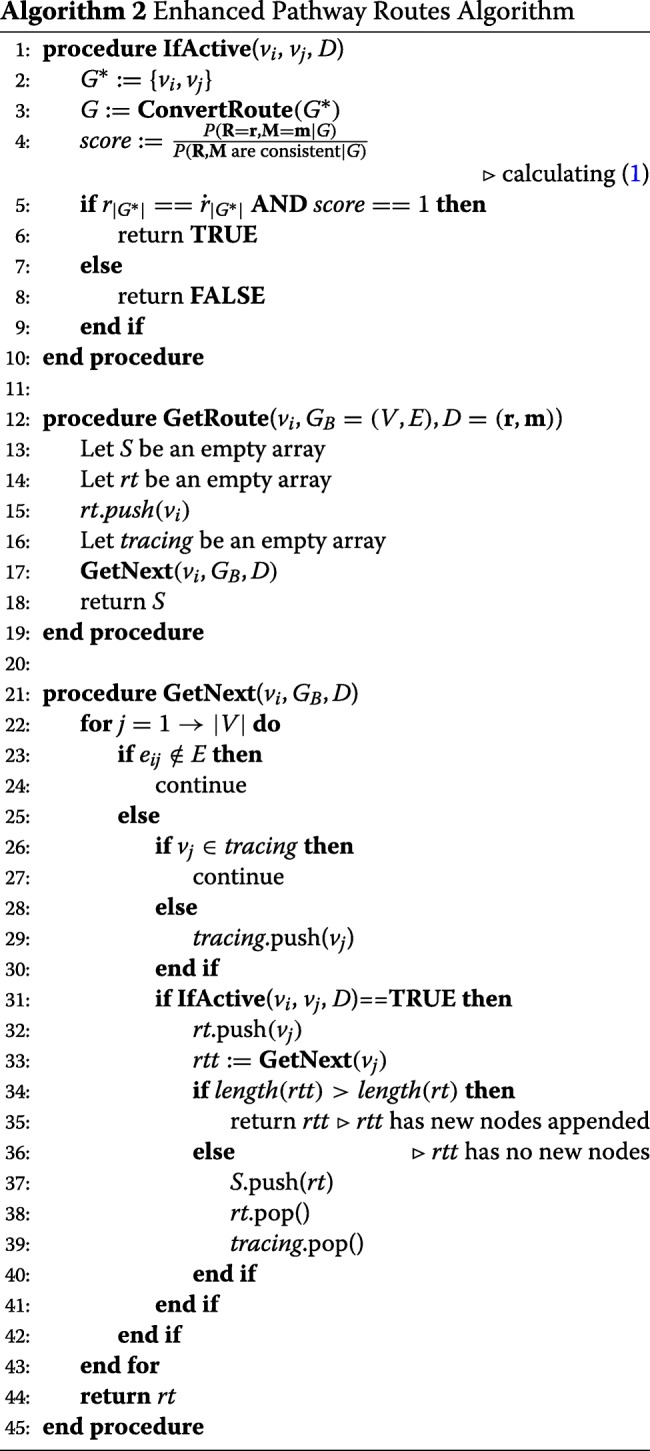


**Fig. 3:**
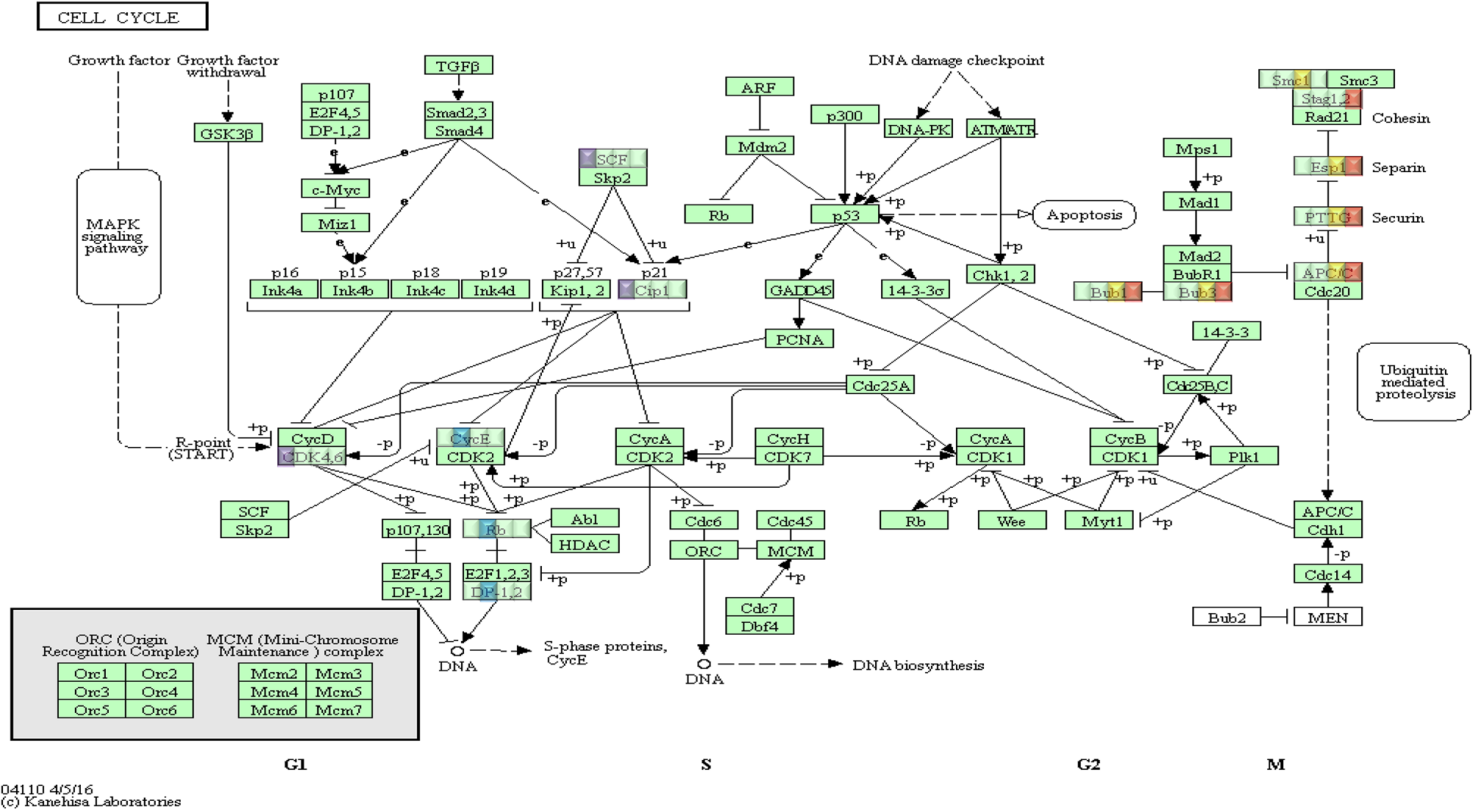
Visualization of identified KEGG Cell Cycle pathway routes. Each node is highlighted with four squares corresponding to G1, S, G2, and M phases. The four phases are respectively colored purple, blue, yellow, orange

Although the Cell Cycle pathway shown in Fig. 2 is from KEGG, it was rendered into a network to emphasize its repeating patterns using Rgraphviz package. Since scientists who use KEGG graphs are not familiar with this rendering, we show in Fig. 3 the original KEGG Cell Cycle graph with routes identified in Table 3 for each cell cycle annotated in different colors, purple for G1, blue for S, yellow for G2 and orange for M. What is noticeable in this color coded display over the original KEGG Cell Cycle graph is that all four routes for G1 through M phases clearly coincide with the nodes mapped by their respective colors. What is also noticeable in this figure is that the color coding of four routes (G1, S, G2 and M) approximately reveal their respective positions from left to right of the graph. This pattern clearly matches that this particular KEGG graph is designed to show the transition of G1 through M left to right as such temporality is actually annotated at the bottom of the original graph. We also note that the yellow and orange color overlay of routes for G2 and M phases lands almost at the same set of nodes. This is expected since the two phases are usually not separable and for that reason they are generally denoted as G2/M phase[24].

**Fig. 4:**
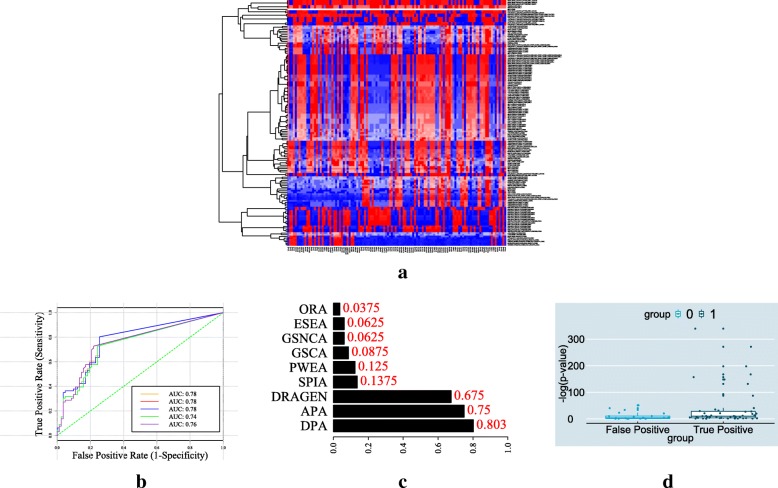
(**a**) The heatmap for Cell Cycle pathway route scores: the columns corresponds to time points and the rows corresponds to pathway routes. Red indicates enhancement while blue indicates suppressiveness. Periodically up and down behavior can be observed as the time flows. Certain route scores behaves distinctly during different phases. (**b**) Receiver operating characteristic (ROC) curve for p53 mutation pathway prediction. The orange, red, blue, green and purple curve corresponds to *t*=0.1,0.3,0.5,0.7,0.9 in (3) respectively. The threshold is picked from [0,1] with a step of 1/10000. (**c**) Proportion of pathways correctly predicted for each pathway analysis tools. (**d**) False positive vs True positive −*log*(*SigScore*) box plot

Lastly, we show the result of performing hierarchical clustering on the identified routes and scores in Fig. 4a. Noticeable in this heat map is the clear consistency between the transition of cell cycling phases and what has been reported in the original publication of the data set [24].

### Comparison with Other Tools on P53 Mutation Dataset

In this section, same pathway analysis [23] on p53 mutation dataset[34] is performed with Deep pathway analysis (DPA). The corresponding results is then compared against the APA[23], ORA[35], GSCA[36], GSNCA[37], ESEA[38], SPIA[15], PWEA[39] and DRAGEN[22].

#### Data Description

The p53 mutation microarray dataset has been widely used as a pathway enrichment analysis benchmark, containing 33 test samples having mutated p53 and 17 wild type control samples. First, the test vs control data is processed with LIMMA[5] using the same parameter settings as [23] and the detected significantly (LIMMA p-value <0.05) Differentially Expressed(DE) genes logFC score is used as **r** data for DPA. Since TP53 is known mutated in this sample, we set *m*=−1 for TP53 and *m*=*NULL* for all others. We attempted to follow the exact same procedure used for the pathway analysis described in [23] as our objective was to make a direct comparison between APA [23] and our work DPA. The pathways having at least one target gene of p53 is labeled as 1 and the other pathways are labeled as 0 where the targets of p53 are obtained from [40]. Unfortunately, both KEGG pathway database and p53 target gene list have been updated since APA has been published. Thus we only used 148 pathways of which 66 labeled as 1 and limited our comparison against the proportion of pathways correctly predicted in [23].

#### Result and Discussion

We use the pathway score, *pScore*, given in (3) to rank the pathways. We calculate the true positive rate and false positive rate in which pathways with scores higher than some threshold is declared to be class 1. By taking different thresholds, ROC curve is obtained and the result is shown in Fig. 4b. The best Area Under Curve (AUC) for DPA is 0.78 and this value is close to 0.8 which has been reported for APA [23]. The proportion of correctly predicted “altered” pathways for each study is shown in Fig. 4c. In this figure, the data for ORA, ESEA, GSNCA, PWEA, SPIA, DRAGEN and APA are directly imported from [23] and included for the comparison purpose. Noticeable in this figure is that DPA reports higher percentage of p53 altered pathways than APA (i.e., 0.80 vs. 0.75). Specifically, DPA predicted 53 out of 66 (0.80) as altered pathways where key known ones such as “Pathways in cancer”, “Jak-STAT signaling pathway”, “Prostate Cancer pathway”, and “p53 signaling pathway” are all included. We note that there are 61 pathways identified as altered by both APA (*DR*≥0.05) and DPA (*pScore*>0). In that regard, the accuracies of both systems can be seen quite comparable. As an alternative comparison study, APA and DPA have been compared using only 132 pathways from the newest version of KEGG, 66 for class 1 (i.e., containing p53 targets) and 66 for class 0 (i.e., containing no p53 target). The results are 0.76 for APA and 0.78 for DPA making DPA outperforms APA by 0.02.

In terms of delivering explanation for the biologists, DPA offers a far greater benefit over APA by presenting the prior knowledge in a manner that biologists are familiar with, i.e., gene regulatory relationships organized into topological pathways. One reason that previous pathway analysis tools fails to work well is because they mainly try to discover perturbed pathways by individual differentially expressed genes instead of gene to gene interactions [23]. Both APA and DPA are newer generation pathway analysis systems which exploits gene-gene interaction relationships in calculating the degree of pathway perturbation, but there is one major difference between APA and DPA in the mechanisms of identifying altered pathways. APA constructs pathway networks dynamically based on gene co-expression whereas DPA uses activation relationship and inhibition relationship as two different forms of prior knowledge. APA measures the perturbation in a pathway by the “similarity” between gene expression test data and control data but DPA measures the “consistency” between expression data and the regulatory relationships encoded into the pathway diagrams.

Lastly, to show the effectiveness of SigScore given in (6), we calculate the −*log*(*SigScore*) for false positive (pathways not having p53 targets but identified) vs true positive (pathways having p53 and identified) and produce a box plot comparison, as shown in Fig. 4d. The Welch two sample *t*-test[41] performed for these two groups of SigScores produces the p-value 0.008634, clearly suggesting that the SigScores for the true positive group are significantly lower than those for the false positive group. This result indicates that SigScore can recognize pathways that acquire a high score by chance.

## Conclusions

We proposed a set of algorithms which given a gene expression data set can compute and score the “perturbed” portion of biological pathways. This method identifies overly regulating routes (or "axes") of pathways by calculating the conditional probabilities of regulatory relationships encoded into Bayesian networks which are constructed from known biological pathways. Our method has been tested with two well-known, publicly available microarray data sets. In our application to the cell cycling microarray data, our method can “recognize” specific portions of pathways clearly revealing cell cycle phase transition with which biologists can easily identify the localized perturbation patterns. We demonstrated through pathway network visualization that our method can clearly reveal how activated pathway routes changes over time and if such pattern change repeats as cell cycling progresses. In our comparison study with APA, our approach demonstrates a comparable accuracy in recognizing perturbed pathways as our method algorithmically identifies isolated sub-network of the pathway as opposed to computing pathway’s perturbation status using enrichment statistics. The name DPA (Deep Pathway Analyzer) originates from the novelty of our method that can deeply recognize perturbed portions of the pathways. Our method of programmatically identifying ”localized” regulating portion of the pathways could pave a new way to carry out future pathway analysis.

## Data Availability

All data analyzed during this study are included in this article.

## Abbreviations

AUC: Area Under Curve
DE genes: Differentially Expressed genes
DPA: Deep Pathway Analyzer
KEGG: Kyoto Encyclopedia of Genes and Genomes
ROC curve: Receiver operating characteristic curve
